# Influence of Elastin-Like Polypeptide and Hydrophobin on Recombinant Hemagglutinin Accumulations in Transgenic Tobacco Plants

**DOI:** 10.1371/journal.pone.0099347

**Published:** 2014-06-10

**Authors:** Hoang Trong Phan, Bettina Hause, Gerd Hause, Elsa Arcalis, Eva Stoger, Daniel Maresch, Friedrich Altmann, Jussi Joensuu, Udo Conrad

**Affiliations:** 1 Department of Molecular Genetics, Leibniz Institute of Plant Genetics and Crop Plant Research (IPK), Gatersleben, Germany; 2 Cell and Metabolic Biology, Leibniz Institute of Plant Biochemistry (IPB), Halle, Germany; 3 Microscopy Unit, Biocenter, University of Halle-Wittenberg, Halle, Germany; 4 Molecular Plant Physiology, University of Natural Resources and Applied Life Sciences, Vienna, Austria; 5 Department of Chemistry, University of Natural Resources and Applied Life Sciences, Vienna, Austria; 6 Department of Plant Cell Biotechnology, Institute of Biotechnology (IBT), Vietnam Academy of Science and Technology (VAST), Hanoi, Vietnam; 7 VTT Technical Research Centre of Finland, Espoo, Finland; Chinese Academy of Medical Sciences, Peking Union Medical College, China

## Abstract

Fusion protein strategies are useful tools to enhance expression and to support the development of purification technologies. The capacity of fusion protein strategies to enhance expression was explored in tobacco leaves and seeds. C-terminal fusion of elastin-like polypeptides (ELP) to influenza hemagglutinin under the control of either the constitutive CaMV 35S or the seed-specific USP promoter resulted in increased accumulation in both leaves and seeds compared to the unfused hemagglutinin. The addition of a hydrophobin to the C-terminal end of hemagglutinin did not significantly increase the expression level. We show here that, depending on the target protein, both hydrophobin fusion and ELPylation combined with endoplasmic reticulum (ER) targeting induced protein bodies in leaves as well as in seeds. The *N*-glycosylation pattern indicated that KDEL sequence-mediated retention of leaf-derived hemagglutinins and hemagglutinin-hydrophobin fusions were not completely retained in the ER. In contrast, hemagglutinin-ELP from leaves contained only the oligomannose form, suggesting complete ER retention. In seeds, ER retention seems to be nearly complete for all three constructs. An easy and scalable purification method for ELPylated proteins using membrane-based inverse transition cycling could be applied to both leaf- and seed-expressed hemagglutinins.

## Introduction

The efficient production of recombinant eukaryotic proteins in native conformations is a major goal for the biotechnological production of therapeutic proteins. This goal can only be achieved at high costs in cell-based production systems such as bacteria and animal cells. Such production platforms are either highly capital-intensive, such as mammalian cell cultures based on secreted proteins, or they require complex and efficient refolding of bacterially expressed proteins from insoluble inclusion bodies. Transgenic plants are promising tools to address the limitations of current production systems for recombinant proteins (for reviews see [1]–[6]). The benefits of plants are the unlimited scale-up potential and relatively low production costs [7]. Notably, the cost of downstream processing steps is generally similar in all recombinant production systems. Downstream costs can account for more than 80% of the overall processing costs [7], [8]. The economical production of plant-made recombinant proteins is therefore limited by two major challenges, inadequate accumulation levels and the lack of efficient and scalable purification methods. The design and production of fusion proteins is a general strategy for overcoming these limitations. Recently, three protein fusion systems, ZERA fusions, hydrophobin (HFBI) fusions and fusions with elastin-like polypeptide (ELP), that allow high intracellular accumulation of recombinant proteins in separate and newly formed storage organelles were described. Additionally, these strategies facilitate the development of specific purification processes [9]–[14]. These three protein fusion systems have very distinct origins; gamma-zein is a plant protein, hydrophobin is a fungal protein, and elastin is of human origin. All three proteins share characteristics that likely cause unique behavior upon plant expression and could cause a significant increase in recombinant protein accumulation. The formation of protein bodies (PBs) induced by both ELP and hydrophobin fusion has recently been investigated using ER-targeted green fluorescent protein (GFP) in transgenic tobacco leaves [12]. An increase in the accumulation and formation of PBs has been shown. An important question is whether this generally holds true for seed expression. The formation of PBs of ELPylated IgG [15] and a slight increase of production levels in seeds have been shown. Another interesting task is to apply these approaches to other therapeutic proteins. ELPylation has been used for the enhancement of expression and purification of several proteins from plants such as antibodies, antigens, cytokines and spider silk proteins (for review see [13]). The ELP fusion strategy is a simple method of purifying recombinant proteins by inverse transition cycling (ITC). The purification process is temperature- and salt-dependent. Increases in salt concentration (NaCl) and temperature result in the formation of insoluble ELP fusion proteins that are separated from the solution by centrifugation at a defined temperature [16]. The insoluble ELP fusion proteins are then re-solubilized in a cool and low-ionic buffer. This purification method was described in 1999 and called centrifugation-based ITC (cITC) [16]. cITC was used to successfully purify antibodies against HIV [15], [17], mycobacterial antigens [14], scFv [18], nanobodies [19], spider silk proteins [20], beak and feather disease virus capsid protein [21], erythropoietin [22] and soluble gp130 [23] from plant cells. However, the cITC process had to be optimized to efficiently enrich every individual protein. Another method to enrich ELP fusion proteins from plants called membrane-based ITC (mITC) was developed and optimized by Phan and Conrad (2011) [24]. Hemagglutinin (HA) and neuraminidase from the avian flu virus have been expressed as ELPylated proteins in plants and purified by this simple and scalable method from leaves [24]–[26].

Influenza viruses continue to be responsible for pandemics [27]. HAs, major antigens of the influenza viruses, are of general interest. Outbreaks of swine flu (H1N1) and avian flu (H5N1) have triggered global concern [28]. Obviously, the proper folding and trimerization of HA antigens requires multiple posttranslational modifications including glycosylation and disulfide bond formation that occur in higher eukaryotic systems. The trimerization could likely result in sufficient antigenicity [29]. Mammalian cell-derived HA trimers induced much higher levels of neutralizing antibodies than similarly produced monomeric HA protein [30]. Plant-generated vaccines focused on HA virus-like particles (VLPs) have been developed [31], and clinical testing is currently underway [32].

To further explore fusion protein strategies in terms of the enhancement of expression and development of scalable purification methods, fusions with hydrophobin and ELP are compared here. Seed-specific expression is included in this evaluation. To assess organ-dependent differences in the formation of induced PBs and the post-translational modification of the recombinant proteins, we analyzed the compartmentalization both in seeds and leaves and took a closer look at the glycosylation of plant-produced HA.

## Materials and Methods

### Generation of plant vectors

**Figure 1:**
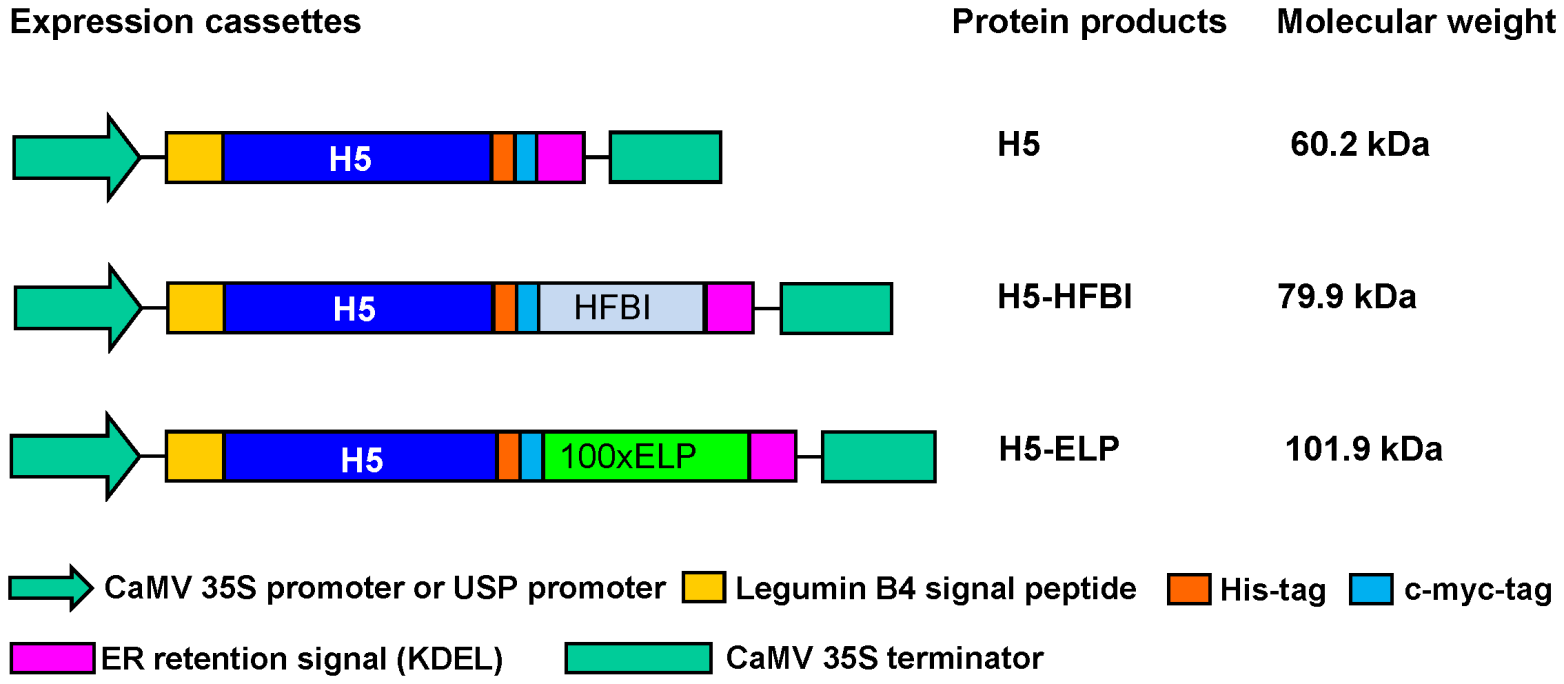
Expression cassettes for hemagglutinin (HA) in plants. HAs were stably expressed in both leaves and seeds under the control of the CaMV 35S and the seed-specific promoters as the naked form (H5), hydrophobin I fusion protein (H5-HFBI) and ELPylated H5 (H5-ELP). All recombinant HAs contained His and c-myc tags for affinity chromatography purification and Western blotting, respectively. The LeB4 signal peptide and KDEL motif were used to ensure ER retention.

The DNA sequence encoding amino acids 2–564 of hemagglutinin subtype 5 (H5) from the A/Hatay/2004/(H5N1) strain (GenBank accession Q5QQ29) was optimized for expression in tobacco plants and synthesized by GENEART AG (Regensburg, Germany). The previously published constructs were used for expression of H5 and H5-ELP under the control of the CaMV 35S promoter [25] (Figure 1), while the TBAG gene in pRTRA-USP-TBAG and pRTRA-USP-TBAG-ELP (unpublished data) was replaced by the synthesized DNA sequence encoding ectodomain H5 (aa 17–520) to generate expression cassettes (pRTRA-USP-H5 and pRTRA-USP-H5-ELP) under the control of a seed-specific promoter with the introduction of sequence coding for a 6xHis tag (Figure 1).

The DNA fragment coding for the TEV cleavage-recognition site, a flexible (GGGS)_3_ linker and the hydrophobin gene was amplified by PCR using extension primers and the pTNS9 vector containing the HFBI coding sequence as a template [33]. The HA1 and H5 coding sequences of the pRTRA-35S-HA1 and pRTRA-USP-H5 vectors were replaced by the PCR product to generate pRTRA-35S-HFBI and pRTRA-USP-HFBI, respectively. The DNA sequence encoding ectodomain H5 (aa 17-520) was cloned into the resulting pRTRA-35S-HFBI and pRTRA-USP-HFBI to form the expression cassettes pRTRA-35S-H5-HFBI and pRTRA-USP-H5-HFBI, respectively.

All expression cassettes in pRTRA vectors were subcloned by *Hin*dIII cleavage into the shuttle vector pCB301-Kan [17], [34].

### Plant transformation

Tobacco was transformed by agroinfection by the leaf disc method described by Horsch and co-workers [35]. The *Agrobacterium tumefaciens* C58C1 strain transformed with the pCB-kan binary vectors was grown overnight in YEB medium containing 50 µg/ml kanamycin, 50 µg/ml carbenicillin and 50 µg/ml rifampicin. Tobacco leaf discs were submerged for 1 h in the agrobacterium culture and then plated on MS medium at 24°C in the dark for another two days. Infected leaf fragments were transferred to MS medium containing 0.2 mg/L α-naphthalene acetic acid, 1 mg/L 6-benzylaminopurine, 50 mg/L kanamycin and 500 mg/L cefotaxim (NBKC medium). Every 10–14 days, leaf discs were transferred to new NBKC medium until plantlets 2–3 cm in length appeared. These plantlets were transferred to MS medium containing 50 mg/L kanamycin [36] and transferred to soil in the greenhouse. The leaves and seeds of these plants were used for Western blot analysis to screen transgenic plants expressing recombinant proteins under control of the CaMV 35S and seed-specific promoters, respectively.

### SDS-PAGE and Western blot

Western blots were performed following the protocol described by Gahrtz and Conrad (2009). In brief, proteins in SDS sample buffer [37] were kept at 95°C for 10 min. The concentration of total soluble protein (TSP) was determined using the Bradford assay. Plant proteins were separated by reducing SDS-PAGE (10% polyacrylamide) and electrotransferred to nitrocellulose membranes. After blocking with 5% (w/v) fat-free milk powder dissolved in TBS (20 mM Tris, 180 mM NaCl, pH 7.8), the membranes were incubated for 2 h at room temperature with a monoclonal anti-c-myc antibody. Antibody binding was detected by the addition of a 1∶2,000 dilution of HRP-conjugated sheep anti-mouse IgG. Each membrane was washed three times between each step with TBS containing 0.5% w/v fat-free milk except for the penultimate (TBS only) and final (phosphate-buffered saline, PBS (137 mM NaCl, 2.7 mM KCl, 10 mM Na_2_HPO_4_, 1.8 mM KH_2_PO_4_, pH 7.4) washes. Antibodies were diluted in TBS with 5% (w/v) fat-free milk powder. The signal was visualized using the enhanced chemiluminescence method (GE Healthcare, UK).

To obtain a rough estimation of the expression level of recombinant proteins, an immunoblotting technique was applied. In general, the samples were serially diluted to achieve band intensities that were similar to the band intensity of a standard protein (^Nt^anti-hTNFa-VHH-ELP [19]) used at a known amount and containing the c-myc tag for Western blot detection. Band intensities were measured by using totalLab Quant software (Nonlinear Dynamics, USA).

#### Protein purification by ITC

ELP fusion proteins were purified using mITC optimized to enrich ELPylated proteins from tobacco leaves [24]. Briefly, frozen *N. tabacum* leaves were homogenized in ice-cold 50 mM Tris-HCl (pH 8.0). The plant extract was then cleared by centrifugation (75,600 *g*, 30 min, 4°C). NaCl was added to the extract up to 2 M. The cold extract was centrifuged again at 75,600 *g* for 30 min and then passed through a 0.22 µm polyethersulfone membrane (Corning, USA) with the temperature maintained at 4°C. The clear extract was warmed to room temperature and passed through a 0.2 µm cellulose acetate membrane using a vacuum pump (Vacuubrand, Germany). The membrane was washed twice with 2 M NaCl to remove contaminating proteins. Ice-cold MilliQ water was then passed through the filter to elute the ELP fusion proteins.

ELPylated H5 from seeds was initially purified using the mITC procedure described above. mITC purification of ELPylated HA from seeds was improved by introduction of ammonium sulfate precipitation before adding NaCl. The following steps were performed as described above.

#### Protein Purification by immobilized metal ion chromatography

Leaf/seed samples were ground with a mortar and pestle in liquid nitrogen. Total protein was extracted in 50 mM Tris buffer (pH 8.0). The extract was clarified by centrifugation (18,000 *g*, 30 min, 4°C) and then filtrated through paper filters. The clear extract was mixed with Ni-NTA agarose resin previously washed twice with water. After mixing for 30 at 4°C, the mixture was applied to a chromatography column. Thereafter, the column was washed with washing buffer (50 mM NaH_2_PO_4_, 300 mM NaCl, 20 mM imidazole, pH 8.0). Recombinant proteins were then eluted from the column with elution buffer (50 mM NaH_2_PO_4_, 300 mM NaCl, 125 mM imidazole, pH 8.0). The protein was concentrated with an iCON Concentrator (Thermo Scientific, USA) with a molecular weight cutoff of 9,000 and stored at −20°C.

#### PNGase F treatment

Purified H5, H5-HFBI and H5-ELP were deglycosylated with the commercial PNGase F enzyme (Catalog No. P0704S; New England Biolabs, Germany). Briefly, 1 µg recombinant purified HA was incubated at 37°C in the presence of PNGase F enzyme for 1 h. The enzyme was inactivated by incubation at 75°C for 10 minutes. After enzyme inactivation, proteins in SDS sample buffer were separated on a 10% reducing SDS-PAGE, blotted and analyzed by Western blot using the anti-c-myc monoclonal antibody as described in detail above.

#### Immunofluorescence and immunogold labeling

Leaves from transgenic and wild-type tobacco plants were embedded in polyethylene glycol (PEG) [38] and in HM20 after high-pressure freeze fixation and freeze substitution [39] for immunofluorescence and immunogold labeling, respectively. Semithin sections of 3 µm thickness mounted on glass slides for immunofluorescence and sections showing silver interference mounted on copper grids for electron microscopy were blocked in 5% BSA in 0.1 M phosphate buffer saline (PBS, pH 7.4). Sections were then incubated with monoclonal mouse anti-c-myc antibody diluted 1∶20 in 5% BSA/PBS at 4°C overnight (fluorescence) or at room temperature for 2 h (electron microscopy). The antibody-antigen reaction was visualized with polyclonal goat anti-mouse serum conjugated to Alexa Fluor 488 or 594 (Invitrogen, UK) for fluorescence microscopy or polyclonal goat anti-mouse serum conjugated to 10 nm gold (British Biocell International, Cardiff, UK) for electron microscopy. Semithin sections of leaves were counterstained by incubation with 0.1 µg/ml 4,6-diamidino-2-phenylindol (DAPI; Sigma-Aldrich, Germany) for 15 min. Micrographs were obtained using a Zeiss Axioimager microscope (Carl Zeiss Microscopy GmbH, Germany) or a LIBRA 120 transmission electron microscope (Carl Zeiss Microscopy GmbH). All micrographs were processed with the Photoshop 12.0.4 program (Adobe, Germany). Seeds expressing H5 alone, H5-ELP and H5-HBFI fusions as well as wild type seeds were fixed, dehydrated and embedded as described [15], [40]. The detection of the signal was performed as described for the leaf sections.

#### N-glycan analysis

The purified HAs were separated by reducing SDS-PAGE in 10% polyacrylamide gels under reducing conditions. Coomassie-stained bands were excised, destained, reduced, carbamidomethylated and digested with trypsin. Tryptic peptides were digested with PNGase A [41]. The released N-linked glycans were then purified using porous graphite carbon spin columns (PGC, Hypercarb; Thermo Scientific) and analyzed by reflectron MALDI-TOF-MS on an Autoflex instrument (Bruker, Germany).

#### Statistical analysis

Statistical analyses were performed using the SigmaPlot software. The Kruskal-Wallis one way analysis of variance on ranks was applied to test the statistical differences between the median values of the three transgenic plant groups. A P value less than 0.01 was defined as a significant difference.

## Results

### Expression of influenza hemagglutinin variants in plants

**Figure 2:**
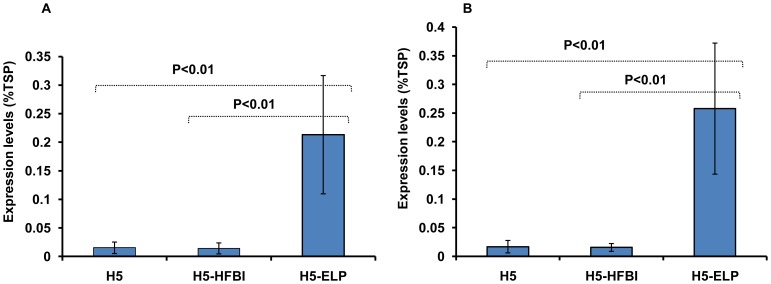
Influence of hydrophobin and elastin-like polypeptide on hemagglutinin accumulation in transgenic plants under the control of either the CaMV 35S promoter (A) or the seed-specific USP promoter (B). Each column shows the mean value of expression level of the transgenic plants, and the error bars indicate the standard deviation. TSP: total soluble protein.

**Figure 3:**
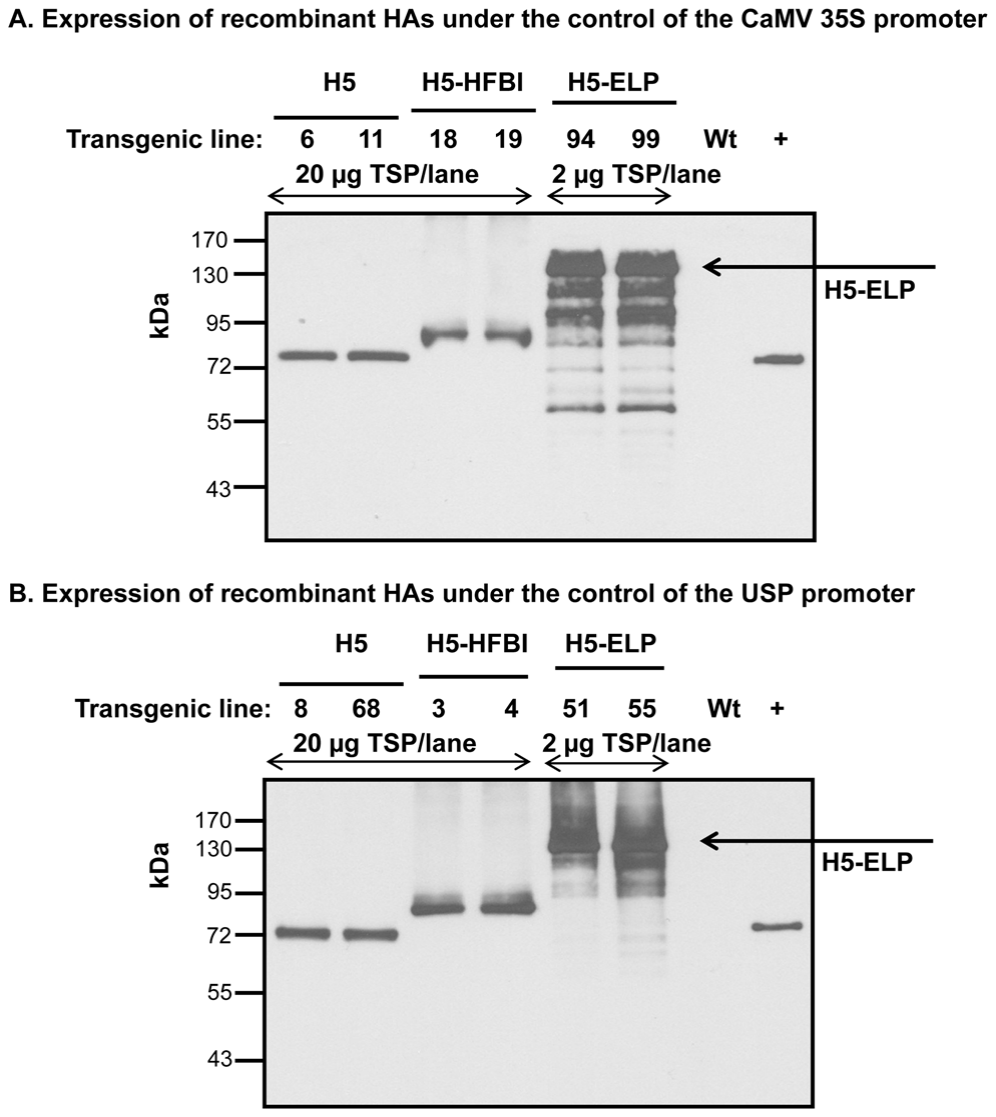
Expression of influenza HAs in transgenic tobacco plants. The extracted proteins from transgenic tobacco leaves (A) or seeds (B) were separated by 10% SDS-PAGE, blotted and detected with anti-c-myc monoclonal antibody followed by horseradish peroxidase-linked sheep anti-mouse IgG as a secondary antibody. “+”: anti-hTNFα-VHH-ELP was used as a Western blot standard [19]; Wt: wild type; TSP: total soluble protein. The numbers refer to independent primary transgenic plants.

The influenza HA is a transmembrane type I protein containing a signal peptide, an ectodomain, a transmembrane domain and a cytoplasmic tail [42]. To efficiently express H5 in plant cells, the sequence encoding the ectodomain (aa 17–520) of the A/Hatay/2004/(H5N1) influenza virus strain was synthesized with optimized codons and cloned in expression cassettes (Figure 1). The H5 protein was recombinantly expressed alone (H5) or as a fusion with hydrophobin (H5-HFBI) or ELP (H5-ELP) under the control of both the CaMV 35S and USP promoters (Figure 1). Each recombinant protein contained the c-myc tag for downstream detection by Western blot, a His tag for purification by immobilized metal ion chromatography (IMAC) and the KDEL motif at the C-terminal end to retain the protein in the endoplasmic reticulum (ER) [43]. The functionality of all binary vectors was validated by assaying the expression of recombinant proteins in transiently transformed *N. benthamiana* leaves and confirmed by Western blot (data not shown). Transgenic tobacco plants were generated by Agrobacterium-mediated leaf disc transformation [35], [36]. Transformants were selected on medium containing selecting antibiotics. Regenerated transgenic plants were screened by Western blot using an anti-c-myc monoclonal antibody. The results are shown in Table 1. To monitor the effect of the hydrophobin and ELP fusions on influenza HA accumulation in transgenic plants, the accumulation levels of ELPylated, hydrophobin fused and untagged H5 in leaves and seeds were determined by SDS-PAGE under reducing conditions and Western blot using an anti-c-myc monoclonal antibody and compared to standard proteins with c-myc tag (anti-hTNFα-VHH-ELP [19]). Expression levels of all transgenic plants according the 6 constructs have been analyzed and presented (see Table S1–S6 in File S1). Statistical analyses (P<0.01, Figure 2) show, that ELPylation strongly enhances the expression levels both in leaves and seeds. The hydrophobin fusion had no significant effect on the accumulation of HA in the ER in both leaves and seeds. H5-ELP reached yields 10-fold higher than that of H5-HFBI and untagged H5 in both seeds and leaves (Tables S1–S6 in File S1, Figure 2, 3). H5, H5-HFBI and H5-ELP proteins accumulated to a maximum of 0.04, 0.05 and 0.5% of the total soluble protein in leaves and 0.04, 0.03 and 0.5% in seeds, respectively (Tables S1–S6 in File S1). We also selected among transgenic lines bearing a single locus insertion of transgene (determined by segregation of the *npt II* gene) the two lines with the highest expression for everyone of the six constructs and compared the expression levels directly by Western analyses (Figure 3) to exclude the influence of multiple insertions. The data again indicate, that only ELPylation causes a strong expression enhancement in leaves and seeds as well. This result is in contrast to previous results presented by Joensuu and co-workers [11]. They reported that the hydrophobin fusion significantly enhanced the accumulation of green fluorescence protein and the enzyme glucose oxidase. The apparent molecular weights shown in Figure 3 are higher than the expected sizes predicted from the polypeptide sequences in Figure 1. This could be explained by the fact that glycosylation influences the running behavior during the electrophoretic separation.

**Table 1:** Transgenic tobacco plants expressing recombinant hemagglutinins.

Transgene	Number of regenerated plants	Number of transgenic plants
**CaMV 35S promoter**		
H5*	25	18
H5-HFBI	51	38
H5-ELP*	55	43
**USP promoter**		
H5	36	25
H5-HFBI	65	39
H5-ELP	91	50

Transgenic plants were screened by Western blot using an anti-c-myc monoclonal antibody. H5: hemagglutinin; H5-HFBI: hemagglutinin fusion with hydrophobin I; H5-ELP: ELPylated hemagglutinin.

*Plants expressing H5 and H5-ELP under the control of the CaMV 35S promoter were described by Phan and co-workers [25].

### Purification of plant-derived recombinant hemagglutinins

**Figure 4:**
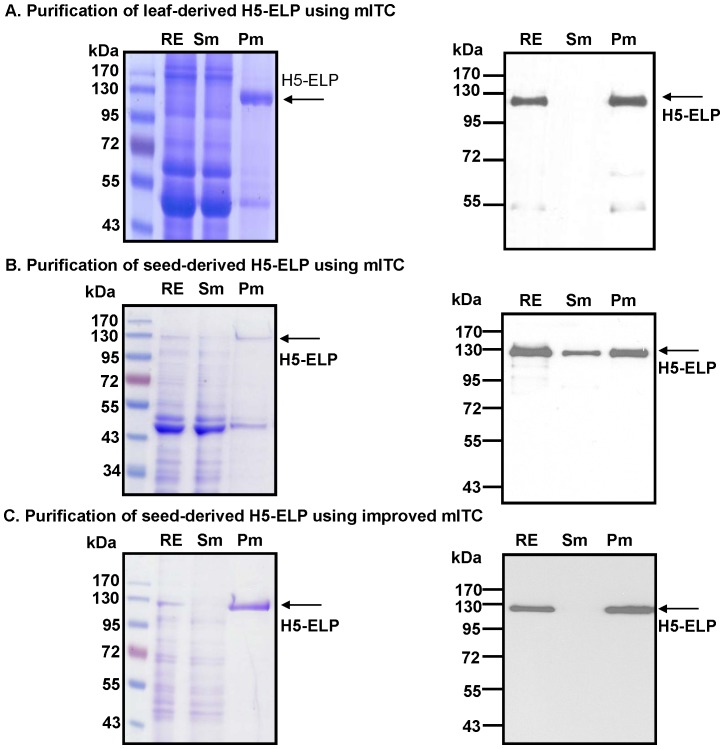
Purification of ELPylated hemagglutinin (H5-ELP) from transgenic leaves and seeds by membrane-based ITC. ELPylated hemagglutinins from leaves (A) and seeds (B) were purified by the standard or improved mITC methods (C) described in the Materials and Methods section. Proteins in the raw plant extract (RE), in the supernatant after passage through a 0.2 µm cellulose acetate membrane (Sm) and in the eluent (Pm) were collected during the mITC purification process and separated by 10% SDS-PAGE. Recombinant proteins were then detected using Coomassie Brilliant Blue staining (left) or an anti-c-myc monoclonal antibody (right).

In this study, ELPylated HAs were purified from leaves by the procedure described by Phan and co-workers [25] (Figure 4A) and from seeds (Figure 4B) by the mITC protocol described by Phan and Conrad (2011) [24]. Coomassie brilliant blue stained gels show that aggregates of H5-ELP were selectively enriched from leaf extract (Pm, Figure 4A) by mITC, while other plant proteins passed through the 0.2 µm cellulose acetate membrane (Sm, Figure 4A, B). The H5-ELP obtained from mITC was highly pure and concentrated (Pm, Figure 4A). In the case of seed-derived H5-ELP, the target protein was enriched (Pm, Figure 4B), but H5-ELP was still detected by Coomassie brilliant blue staining in the flow through (Sm, Figure 4B), and the purified eluted protein was contaminated with plant proteins (Pm, Figure 4B). This result shows that the mITC procedure optimized for purification of leaf-derived HA was not suitable for the purification of ELPylated HA from seeds. Therefore, the mITC method was adapted to purify seed-derived ELPylated HA. We observed that seed extracts were contaminated with oils. To remove plant oils, proteins were precipitated by ammonium sulfate and then solubilized in Tris buffer (see the Materials and Methods section). The purification was proceeded as in the standard mITC. The Coomassie brilliant blue-stained gel showed that seed-derived ELPylated HA was selectively enriched from seed extract with high purity (Pm, Figure 4C). Furthermore, the target protein was not detected by Coomassie staining and Western blot in the flow through (Sm, Figure 4C). The Western blot results shown in Figure 4 indicate that H5-ELP, with the expected size of approximately 100 kDa, was successfully purified from leaves (Pm, Figure 4A) and seeds (Pm, Figure 4B, C). Only a small portion of the target protein or no target protein was detected in the Sm fraction (Sm, Figure 4A or Sm, Figure 4C, respectively) after performing the optimized procedure.

**Figure 5:**
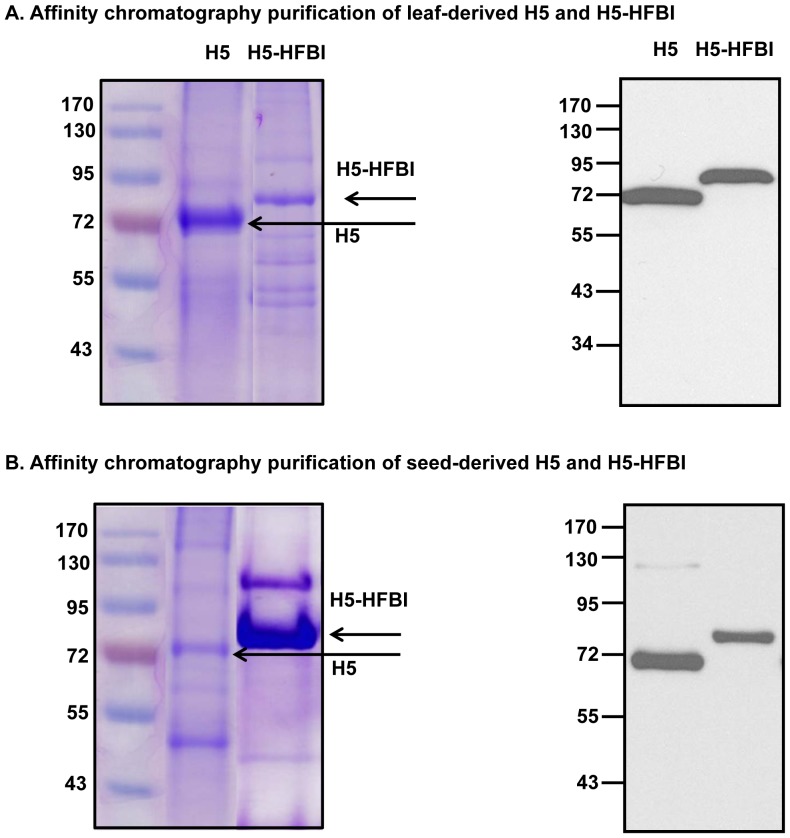
Purification of non ELPylated HA from transgenic leaves and seeds by His tag-based affinity chromatography. Non ELPylated HAs from leaves (A) and seeds (B) were purified by the IMAC method described in the materials and methods section. Purified proteins were separated by 10% SDS-PAGE and then detected using Coomassie Brilliant Blue staining (left) or by an anti-c-myc monoclonal antibody (right).

Hydrophobins are small surface-active fungal proteins that can be purified using a surfactant-based aqueous two-phase system (ATPS; [11], [44]). The presence of the hydrophobin tag did not significantly enhance the accumulation of HA in the ER in both leaves and seeds in comparison with pure HA (Figure 2, 3). Therefore, the purification of hydrophobin fusion HAs using ATPS was not included in this study. Instead, H5-HFBI and pure H5 from leaves and seeds were purified by IMAC based on the C-terminal 6x histidine tag (Figure 1) to obtain material for further characterization. Both HAs were analyzed by Coomassie blue stain and Western blot using an anti-c-myc monoclonal antibody (Figure 5).

### Subcellular localization of recombinant hemagglutinins in leaves and seeds of transgenic tobacco plants

**Figure 6:**
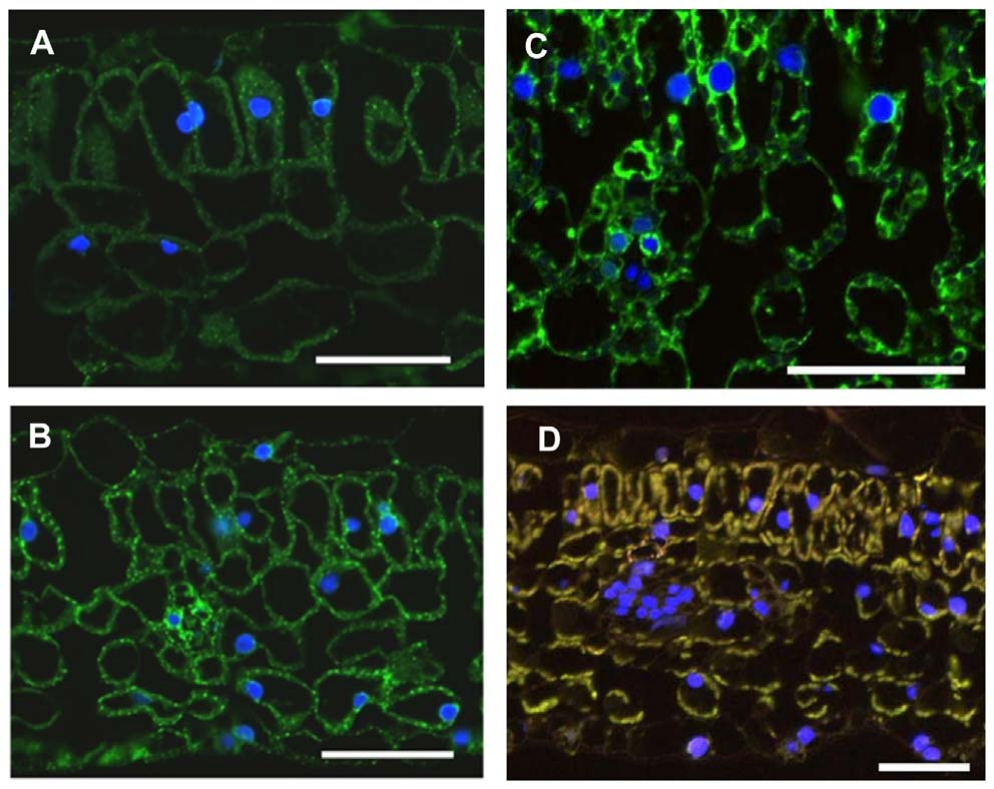
Immunofluorescence analysis of recombinant HAs in plant leaves. Leaves were fixed, embedded in PEG and sectioned. Recombinant HAs were immunodecorated with an anti-c-myc monoclonal antibody followed by incubation with secondary antibody (anti-mouse-IgG conjugated with AlexaFluor488) and counterstaining with DAPI. A. H5; B. H5-HFBI; C. H5-ELP; D. wild type. Bars represent 50 µm.

To monitor the localization of recombinant HAs in transgenic plants, leaves expressing untagged H5, H5-ELP and H5-HFBI were analyzed by fluorescence and electron microscopy *via* c-myc tag detection. Fluorescence microscopy showed that strong fluorescent signals (green color) were detected in leaves expressing recombinant H5 (Figure 6A), H5-HFBI (Figure 6B) and H5-ELP (Figure 6C) compared with the negative control (Figure 6D). Fluorescent signals indicated that recombinant HAs were distributed within the cytoplasm, mainly around nuclei which were stained by DAPI (blue color). Therefore, we hypothesized that recombinant HAs are localized in the ER. Electron microscopy was applied to address this question.

**Figure 7:**
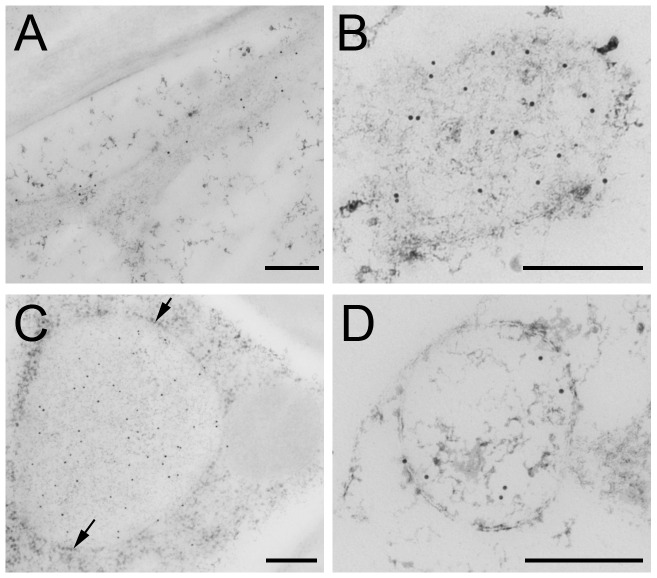
Localization of recombinant hemagglutinins in leaves visualized by electron microscopy. Thin leaf sections mounted on copper grids were probed with monoclonal mouse anti-c-myc antibody followed by the goat anti-mouse-IgG conjugated to 10 nm gold particles. A and B. H5; C. H5-ELP; D. H5-HFBI. The membrane of immunodecorated PB is surrounded by ribosomes (arrows, C). Bars represent 250 nm.

Electron micrographs showed the distribution of untagged H5 that was mainly found in the ER lumen (Figure 7A). In addition, H5 labelling was occasionally detected in PB at a size of 231.68±136.73 nm (n = 10) (Figure 7B). In contrast to H5, the H5-ELP fusion was predominantly detected in the bigger PBs that were typically 835±323.36 nm (n = 35) (Figure 7C). The membrane surrounding the PBs was decorated with ribosomes (arrows, Figure 7C). This result suggested that ELP-induced PBs were derived from the ER membrane and distributed in the cytoplasm as a novel organelle. When H5 was fused to hydrophobin, PBs were also observed (Figure 7D); however, they were more abundant than those observed in the leaves expressing untagged H5 and less abundant than those observed in H5-ELP leaves (Figure 7). H5-HFBI bodies were 426.21±122.44 nm (n = 17) in size.

**Figure 8:**
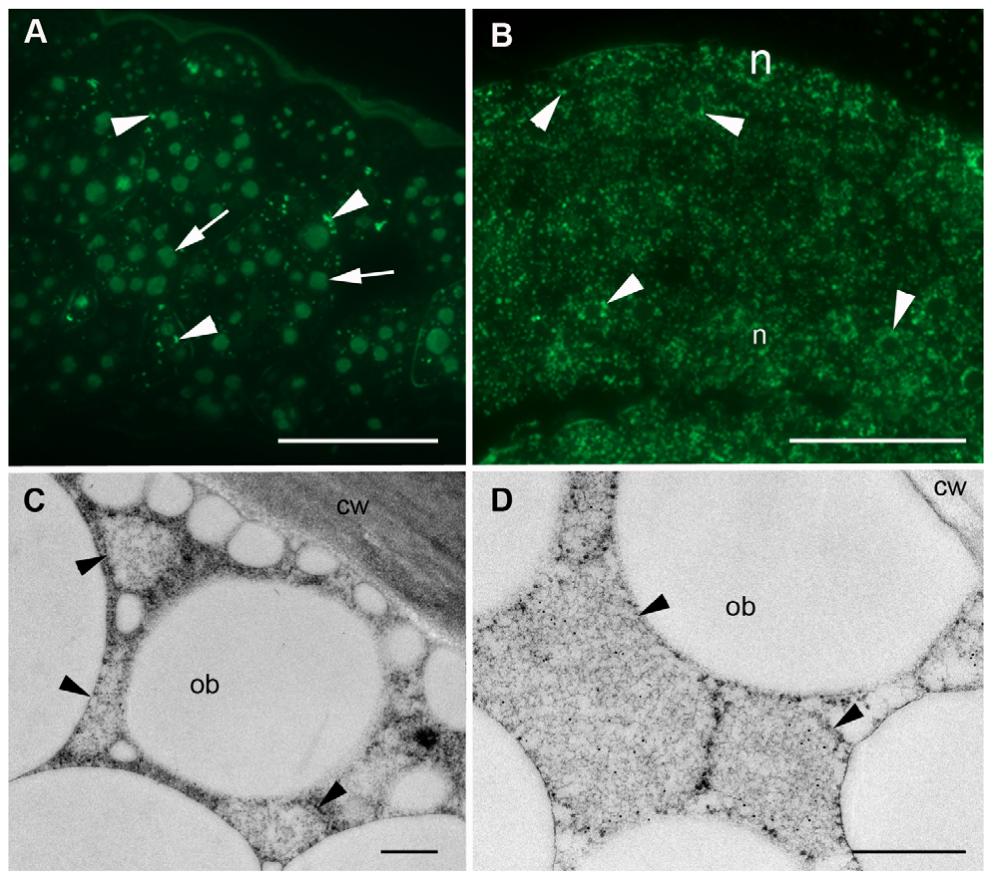
Localization of hemagglutinin-ELP fusions in tobacco seeds. A, B. Fluorescence microscopy.C, D. Electron microscopy.A, C. Endosperm. B, D. Embryo. Note the ELP bodies (arrowheads, A, B) and those that are loosely packed (arrowheads, C, D). Cell wall (cw), oil bodies (ob), protein storage vacuole (arrows), nucleus (n). Bars 50 µm (A, B), 1 µm (C, D).

HA-derived PBs have been observed in transgenic tobacco seeds. When expressed alone, H5 was found within the protein storage vacuoles (PSV) in both the endosperm and the embryo (Figure S1A). H5-ELP fusions formed PBs both in the endosperm and the embryo cells (Figure 8). In the endosperm cells, ELP bodies have the same appearance as observed when fused to antibody chains [15]. Thus, they are irregular in size and shape (Figure 8A) and have a flocular, loosely packed content (Figure 8C). As for the embryo, cells contain a high number of ELP bodies, more abundant than that observed in the antibody fusions [15]. They exhibit the same appearance in endosperm cells and form large masses found within the cytoplasm (Figure 8C, D). No clear labelling was observed in the apoplast or any other compartment of endosperm or embryonic cells apart from a weak nonspecific signal within the crystalloids of the PSVs (Figure 8). PBs observed after ELPylation were not detected in wild-type seeds (Figure S1B).

**Figure 9:**
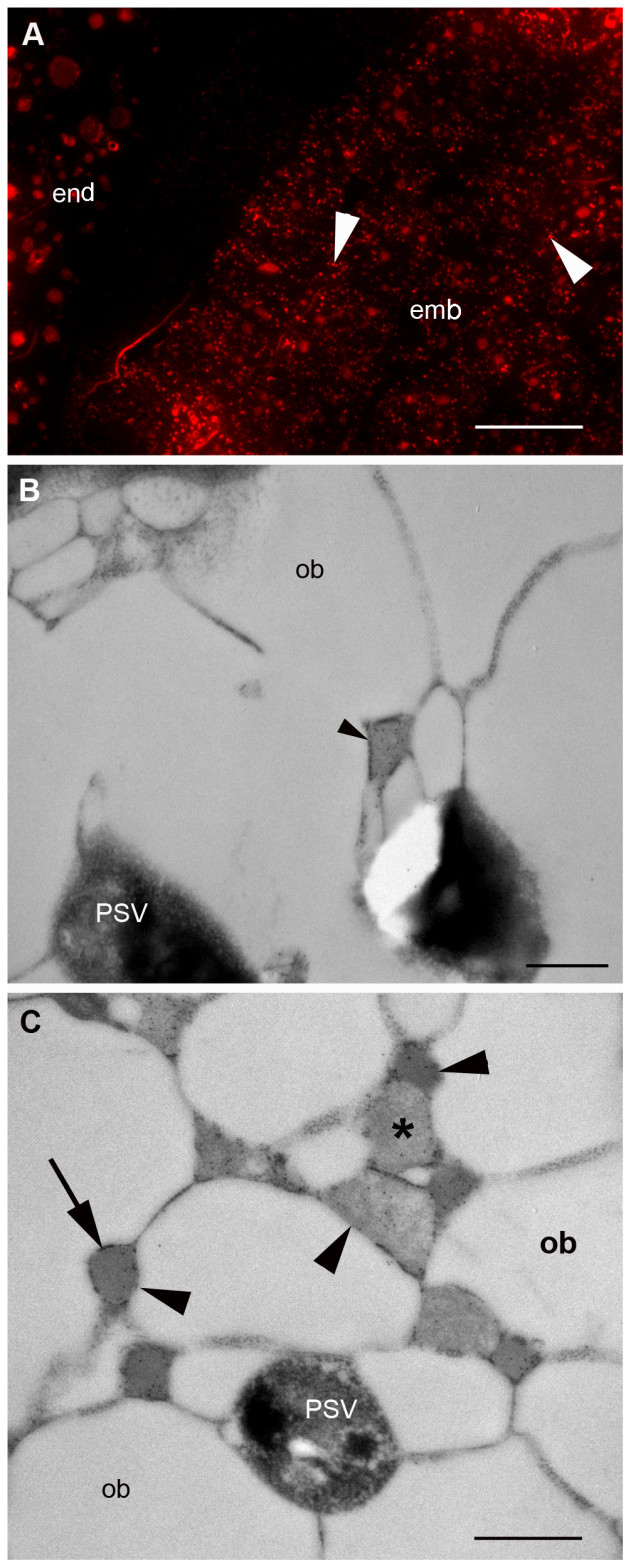
Localization of hemagglutinin-hydrophobin I fusions in tobacco seeds. A. Fluorescence microscopy. B, C. Electron microscopy. B. Endosperm. C. Embryo. Scarce hydrophobin bodies in the endosperm (arrowheads, A, B). Abundant hydrophobin bodies in the embryo cells (arrowheads, A, C). Hydrophobin bodies show non-uniform electron density (*, C). Endosperm (end), embryo (emb), protein storage vacuole (PSV), ribosomes (arrow). Bars 25 µm (A), 0.5 µm (B, C).

HA-hydrophobin bodies were found both in the endosperm and the embryo, although in the endosperm they were rather scarce (Figure 9A). In contrast to ELP, hydrophobin bodies are regular in size and on average much smaller (∼300 nm). These bodies are tightly packed with material of medium electron density (Figure 9B, C) and are most likely ER derived because their membrane appears to be decorated with ribosomes (Figure 9B, C). Some of the hydrophobin bodies, another phase of a loosely packed material, were also labelled with anti-c-myc antibody (Figure 9C). No significant labelling was found in the PSVs or any other compartment of the cell. Hydrophobin bodies were not present in wild-type seeds (Figure S1B).

### Glycosylation profiling of plant-derived hemagglutinins

**Figure 10:**
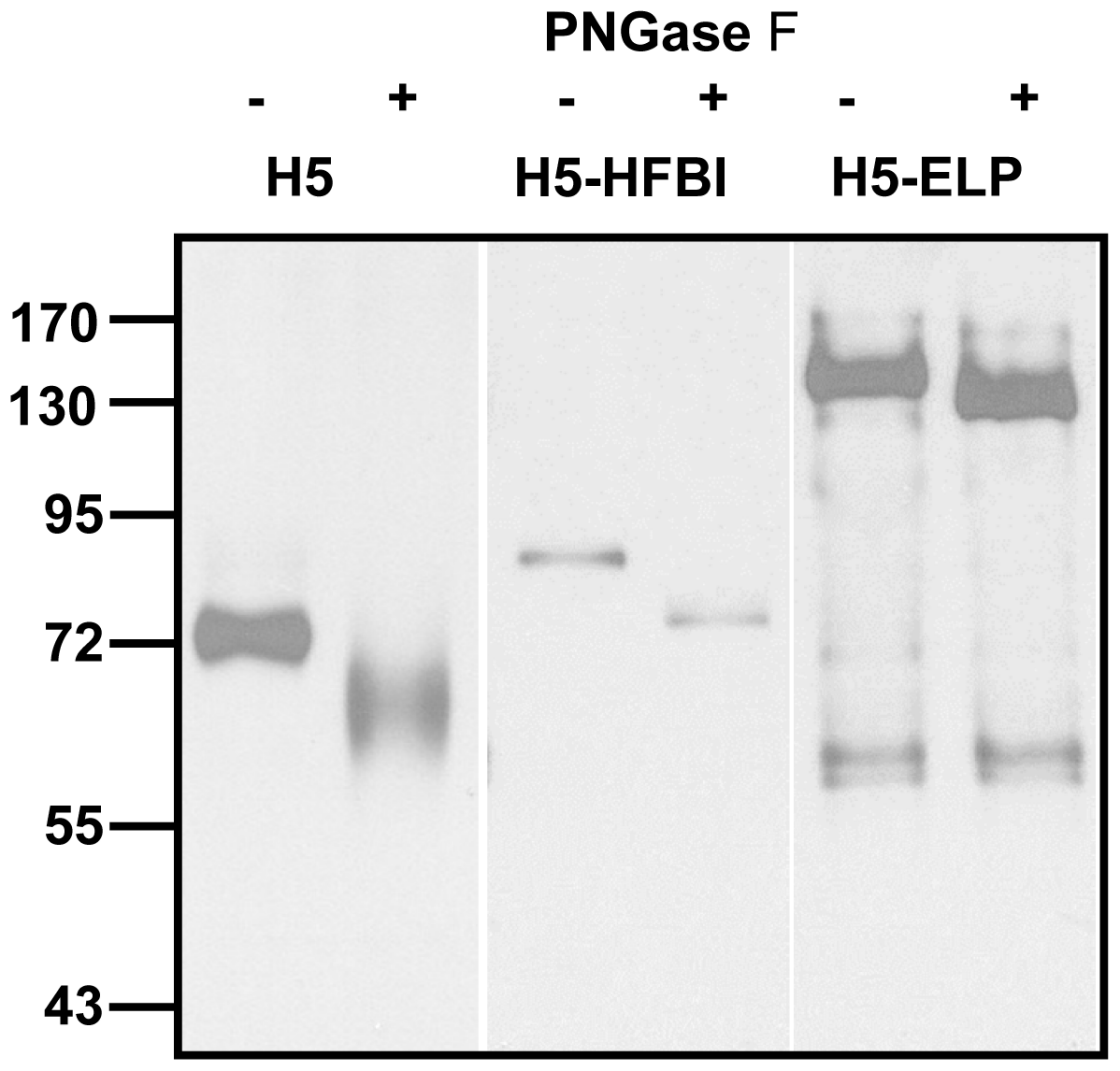
Western blot analysis of purified HAs treated/untreated with PNGase F. Purified HAs from leaves were deglycosylated using the commercial PNGase F enzyme described in the Materials and Methods section. PNGase F-treated and untreated proteins were then separated in 10% SDS-PAGE. Recombinant proteins were detected using an anti-c-myc monoclonal antibody. “−” and “+” indicate PNGase F-untreated and treated samples, respectively.

Influenza HA contains *N*-linked oligosaccharides [45]. Amino acid sequence analysis of the HA of the A/Hatay/2004/(H5N1) strain has predicted six potential *N*-linked glycosylation sites (Asn10, 11, 23, 154, 165 and 286). The glycosylation of recombinant HAs was confirmed by the digestion of purified H5, H5-HFBI, and H5-ELP using the commercial PNGase F enzyme that removes *N*-linked glycans between *N*-acetylglucosamine (Gn) and asparagine residues from glycoproteins. Deglycosylation of plant-derived HAs was visualized by Western blot analysis using an anti-c-myc antibody (Figure 10). Immunodetection revealed that, after removal of the *N*-glycans, the apparent molecular weights of the recombinant HA proteins shifted to the expected molecular weight calculated using the amino acid sequence (Figure 10). This result confirmed that recombinant HAs were post-translationally modified by the addition of *N*-linked glycans.

To further investigate the *N*-linked glycans of the recombinant proteins, HAs purified from leaves and seeds were separated by SDS-PAGE (Figure 4, 5). The bands were excised and digested with trypsin. Mass spectrometry analyses revealed that carbohydrates bound to leaf-derived H5 and H5-HFBI contained 70–72% oligo-mannose type (OMT), with Man_7_GnGn being the most dominant form. Twenty-seven to thirty percent of the glycoforms were complex type (CT) *N*-glycans with typical plant glycans containing fucose and xylose (Table 2). These results indicate that the KDEL sequence-mediated retention of H5 and H5-HFBI in the ER of leaf cells was efficient but not complete for all constructs. Leaf-derived H5 and H5-HFBI were not completely retained in the ER. In contrast, H5-ELP contained only the OMT form; the most abundant glycoforms were Man_7_GnGn (72.32%) and Man_8_GnGn (27.68%). No CT glycans were detected in this case (Table 2). This result indicates that the ELP fusion tag efficiently retained the recombinant H5-ELP in the ER. Interestingly, H5 and H5-ELP accumulating in seeds contained only the OMT form or a very small proportion of the CT glycan form (6.32%, in the case of H5-HFBI) (Table 2).

**Table 2:** Glycosylation profile of recombinant HAs from leaves and seeds.

Recombinant protein	Glycoform (%)				
	Man_6_GnGn	Man_7_GnGn	Man_8_GnGn	GnGnX	GnGnF
**Leaf-derived HA**					
H5		64.08	5.91	25.50	4.50
H5-HFBI		55.12	17.47	27.41	
H5-ELP		72.32	27.68		
**Seed-derived HA**					
H5		75.56	24.44		
H5-HFBI	10.22	61.42	22.04	6.32	
H5-ELP	3.93	44.74	51.33		

Man_6, 7, 8_: Mannose _6, 7, 8_; Gn: *N*-acetylglucosamine; X: Xylose; F: Fucose.

## Discussion

The expression of foreign proteins using plants is a promising method for the safe and low-cost production of antigens for vaccination purposes. However, its widespread use is curtailed by problems related to expression levels, antigen purity and proper post-translational modification which are important for induction of robust immune responses. Currently, ELP and hydrophobin fusion strategies were among the most attractive ways to enhance target protein accumulation in transgenic plants and to provide a simple means for the purification of recombinant proteins (for reviews see [11], [13].

In this study, the major antigen of avian influenza A virus, HA [46], was expressed with C-terminally fused ELP and HFBI. We show here that C-terminal fusion of ELP to HA resulted in increased accumulation of H5-ELP in both leaves and seeds under control of either the constitutive CaMV 35S or the seed-specific USP promoter in comparison to the untagged H5 and H5-HFBI (Figure 2, 3). Our results are consistent with previous studies that reported that expression levels of ELPylated-proteins, such as antibodies [15], [17], anti TNFα nanobodies [19], human interleukin and spider silk proteins [20], [47] and vaccines [14], [25], were higher than those of proteins without ELP fusions. In general, accumulation enhancement by ELPylation was dependent on the specific protein and ranged between two- and 100-fold (for review see [13]). In contrast to ELP, HA fusion with hydrophobin did not significantly enhance the expression level compared to untagged H5, whereas hydrophobin-fused GFP increased two [11] or three-fold in concentration [12] in comparison to untagged GFP. This may be explained by the high accumulation of GFP itself in plant cells (18% and 0.3% of TSP in transiently and stably transformed leaves, respectively [11], [12], while HA accumulated in transgenic plants at a rather low level (0.04% TSP). Again, the enhancement of the expression levels of recombinant proteins in plants caused by hydrophobin is likely depending on the protein fused to HFBI.

In the actual study, we found that increasing expression levels of ER-targeted recombinant proteins are highly related to the presence of novel PBs in the leaves and seeds of transgenic tobacco. ER-targeted pure HA could induce such protein particles in leaves, but they were very rare and small. Novel protein particles containing H5-HFBI were more frequent and larger than those of the H5 alone, whereas H5-HFBI showed only a slightly enhanced accumulation compared to untagged H5 (Figure 7B, D). In contrast to the cases mentioned above, PBs containing H5-ELP were much bigger and formed as novel organelles located in the cytoplasm. Novel PBs induced by HFBI and ELP in leaves were previously reported by Conley and co-workers [48] and by Floss and co-workers for tobacco seeds [15]. The question of whether PB formation is only a result of increased accumulation remains open.

Enhancement of the accumulation of HAs by ELP fusion results in a higher concentration of the ELPylated target proteins in the initial aqueous extraction. Higher levels of ELP-fused proteins facilitate subsequent purification of ELPylated proteins by ITC [49], [50]. ELP fusion proteins could be purified by the ITC method, which is based on precipitation of ELPylated proteins. Micrometer-sized aggregates of ELPylated proteins were collected/harvested by centrifugation or through a 0.2 µm membrane, which are called centrifugation or membrane-based ITC, respectively [24], [51]. We previously reported that avian flu antigens were successfully enriched using an adapted membrane-based ITC method from stably [24] or transiently transformed leaf materials with an efficient recovery rate [25]. In this study, ELPylated hemagglutinin was successfully purified from transgenic seeds using the same mITC procedure for leaf material (Figure 4). However, the target protein was still detected in the flow through after passing through a 0.2 µm membrane (Figure 4B). This result indicates that ELPylated HA from seeds was not completely recovered. An improved mITC method using ammonium sulfate to precipitate proteins and simultaneously remove plant oils was applied to completely recover ELPylated HA from transgenic seeds, suggesting that oils in the protein extract may affect the ability to retain precipitated ELPylated HA on the membrane surface. Our results indicate that ELPylated proteins can be purified from different plant tissues by a simple and inexpensive purification method. Purified ELPylated proteins retain their functionality, such as enzymatic activity [24], the antigen-binding activity of ELPylated antibodies [15] or the receptor-binding activity of ELPylated HA [25]. The low immunogenicity [14], [25] and biocompatibility [52], [53] of ELPs are promising factors in the further application of ELPylation technology for the production and purification of therapeutic proteins for human and veterinary medicine.

In general, glycoproteins are synthesized by ribosomes associated with the ER membrane, translocated into the ER lumen, and trafficked from the Golgi apparatus (GA) toward the plasma membrane and the apoplastic space along the secretory pathway. *N*-linked glycosylation is co-translationally performed on the glycoprotein consensus sequence N-X-T/S (for a review see [54]). *N*-linked glycans of glycoproteins undergo several maturation steps including glycose trimming and mannose addition to form high mannose-type glycans in the ER. *N*-linked glycans are then transported and modified further in the GA from *cis* to medial and *trans* cisternae to form typical plant glycans with the addition of xylose and fucose to the core glycans. In this study, the KDEL motif was used to retain recombinant proteins in the ER [43]. The *N*-linked glycan profiles of these recombinant proteins showed that leaf-derived H5 and H5-HFBI predominantly possess OMT glycans (70–72%) that supposedly matured in the ER compartment and 27–30% CT glycans with typical plant glycans (GnGnX and GnGnF) (Table 2). The abundance of OMT and lower abundance of CT in untagged HA has been reported in previous studies of KDEL-tagged H1 and H5 expressed in transiently transformed plants [55], [56]. In contrast to these studies, we found that H5-ELP exclusively harbored OMT glycans (Table 2). These results suggested that cellular trafficking of H5, H5-HFBI and H5-ELP may be different or the fusion partner could have a shielding effect. H5-ELP is preferentially located in the ER-derived PBs, whereas portions (approximately 70%) of leaf-derived H5 and H5-HFBI are preferentially deposited in the ER-derived PBs. Another portion (approximately 30%) may be trafficked to the GA. Munro and Pelman (1987) reported that KDEL-fused proteins are trafficked into the GA and then back into the ER [57]. Otherwise, the presence of CT glycans in seeds is below 10%. It seems that there is less “escape” of recombinant proteins from the ER. The glycosylation pattern plays an important role for the quality and functionality of plant-produced proteins. From this general point it is important to note, that fusion protein strategies do not only enhance expression levels and purification, but also influence posttranslational modifications due to modified intracellular sorting.

The different subcellular localizations of recombinant HAs were visualized by transmission electron microscopy. The distribution of HAs was consistent with the *N*-linked glycan profiles. We found that the frequency of PB formation and their sizes were dependent on the expression level of recombinant proteins in plant cells. Leaf-derived H5-ELP was predominantly stored in large PBs (diameter 800 nm). H5-HFBI was also deposited in PBs; however, H5-HFBI-induced PBs were much smaller than those of H5-ELP and less abundant. In contrast, H5 alone was located in tubular ER structures and occasionally detected in PBs.

PB formation induced by HA alone suggests that PB formation is not only dependent on ELP or HFBI tags. However, the presence of these tags enhanced the expression levels of recombinant proteins and also increased the frequency of the appearance of PBs. There may be a threshold value for PB formation as noted by Gutierrez and co-workers. They estimated a threshold value of 0.2% TSP [12].

The PBs containing H5-ELP and the absence of CT glycans suggested that this recombinant protein did not arrive in the GA, but is stored in the PBs in the cytoplasm without contact with the GA enzymes that transfer xylose and fucose glycans into core glycans, while H5 and H5-HFBI are likely partially trafficked into the GA and then transported back to the ER.

In summary, in this study we showed that fusing H5 to either HFBI or ELP and ER targeting can induce PBs in leaves as well as in seeds. ELPylation can cause a significant enhancement of expression in both leaves and seeds. In addition, an easy and scalable purification method for ELPylated proteins such as mITC that was previously shown with high recovery efficiency of leaf [24], [25] and seed (in this study) -derived recombinant proteins could be scalably applied to hemagglutinins in the future.

## Supporting Information

Figure S1 **Localization of hemagglutinin in tobacco seeds by immunofluorescence microscopy.** A. Cross-section of transgenic seeds expressing H5. Note the clear signal within the PSVs (arrows) in both the endosperm and in the embryo. B. Negative control using wild type seeds. No labelling was found within any cell compartment in either the embryo or the endosperm. end, endosperm; emb, embryo. Bars represent 20 µm.
(TIF)

File S1 **Table S1–S6.**
(DOCX)
