# Supplementary figures and images for: Influence of Elastin-Like Polypeptide and Hydrophobin on Recombinant Hemagglutinin Accumulations in Transgenic Tobacco Plants
Source: PLoS One. 2014 Jun 10;9(6):e99347. doi: 10.1371/journal.pone.0099347 (PMC4051685; doi:10.1371/journal.pone.0099347)

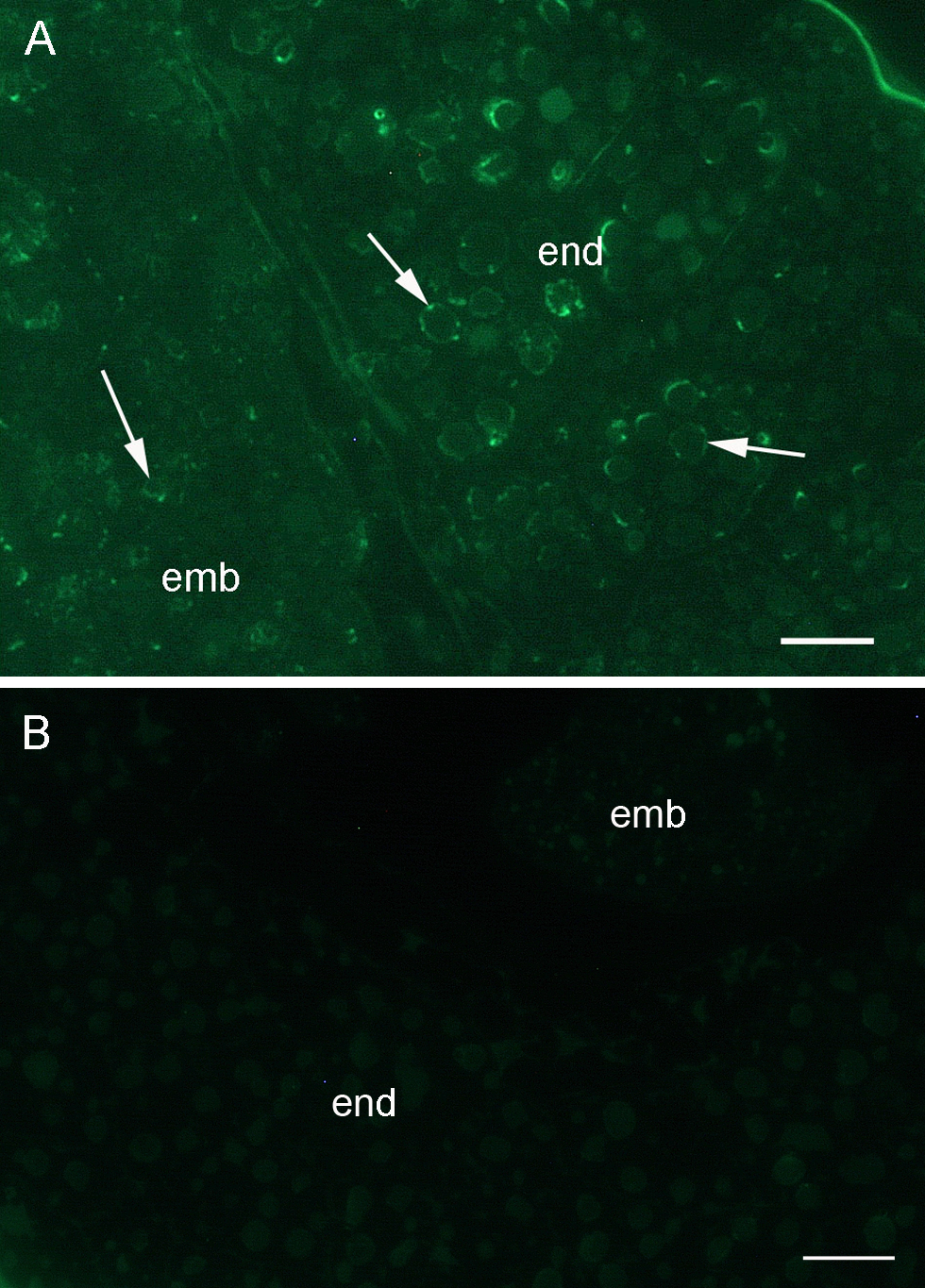

Supplement: Figure S1 — Localization of hemagglutinin in tobacco seeds by immunofluorescence microscopy. A. Cross-section of transgenic seeds expressing H5. Note the clear signal within the PSVs (arrows) in both the endosperm and in the embryo. B. Negative control using wild type seeds. No labelling was found within any cell compartment in either the embryo or the endosperm. end, endosperm; emb, embryo. Bars represent 20 µm. (TIF) [file pone.0099347.s001.tif]
