# Supplementary material for: Influence of Elastin-Like Polypeptide and Hydrophobin on Recombinant Hemagglutinin Accumulations in Transgenic Tobacco Plants
Source: PLoS One. 2014 Jun 10;9(6):e99347. doi: 10.1371/journal.pone.0099347 (PMC4051685; doi:10.1371/journal.pone.0099347)
Supplement: File S1 — Table S1–S6. (DOCX) [file pone.0099347.s002.docx]

Table S1. H5 accumulation levels in transgenic tobacco seeds

| **Number** | **Transgenic line** | **Expression level (%TSP)*** |
| --- | --- | --- |
| 1 | 64 | 0.0008 |
| 2 | 65 | 0.0008 |
| 3 | 19 | 0.0013 |
| 4 | 17 | 0.0014 |
| 5 | 26 | 0.0017 |
| 6 | 67 | 0.0060 |
| 7 | 82 | 0.0123 |
| 8 | 81 | 0.0140 |
| 9 | 83 | 0.0140 |
| 10 | 89 | 0.0146 |
| 11 | 24 | 0.0147 |
| 12 | 86 | 0.0149 |
| 13 | 13 | 0.0155 |
| 14 | 25 | 0.0158 |
| 15 | 55 | 0.0180 |
| 16 | 14 | 0.0188 |
| 17 | 84 | 0.0233 |
| 18 | 6 | 0.0240 |
| 19 | 77 | 0.0253 |
| 20 | 80 | 0.0253 |
| 21 | 57 | 0.0297 |
| 22 | 78 | 0.0307 |
| 23 | 68 | 0.0324 |
| 24 | 66 | 0.0329 |
| 25 | 8 | 0.0354 |

* The quantity of recombinant protein (H5) present in each sample was calculated using the standard protein (anti-hTNFα-VHH-ELP [19]).

**Table S2. H5-HFBI accumulation levels in transgenic tobacco seeds**

| **Number** | **Transgenic line** | **Expression level** [%**TSP**]* |
| --- | --- | --- |
| 1 | 34 | 0.0059 |
| 2 | 45 | 0.0061 |
| 3 | 49 | 0.0061 |
| 4 | 52 | 0.0063 |
| 5 | 24 | 0.0093 |
| 6 | 2 | 0.0095 |
| 7 | 25 | 0.0097 |
| 8 | 5 | 0.0100 |
| 9 | 53 | 0.0103 |
| 10 | 37 | 0.0110 |
| 11 | 22 | 0.0112 |
| 12 | 43 | 0.0115 |
| 13 | 58 | 0.0116 |
| 14 | 29 | 0.0124 |
| 15 | 23 | 0.0124 |
| 16 | 33 | 0.0125 |
| 17 | 8 | 0.0126 |
| 18 | 64 | 0.0128 |
| 19 | 57 | 0.0135 |
| 20 | 55 | 0.0135 |
| 21 | 13 | 0.0140 |
| 22 | 76 | 0.0141 |
| 23 | 65 | 0.0146 |
| 24 | 7 | 0.0160 |
| 25 | 28 | 0.0164 |
| 26 | 35 | 0.0180 |
| 27 | 70 | 0.0182 |
| 28 | 20 | 0.0194 |
| 29 | 71 | 0.0195 |
| 30 | 12 | 0.0197 |
| 31 | 15 | 0.0211 |
| 32 | 6 | 0.0216 |
| 33 | 61 | 0.0244 |
| 34 | 59 | 0.0246 |
| 35 | 4 | 0.0257 |
| 36 | 3 | 0.0279 |
| 37 | 19 | 0.0281 |
| 38 | 10 | 0.0282 |
| 39 | 14 | 0.0303 |

^*^ The quantity of recombinant protein (H5-HFBI) present in each sample was calculated using the standard protein (anti-hTNFα-VHH-ELP [19]).

Table S3. H5-ELP accumulation levels in transgenic tobacco seeds

| **Number** | **Transgenic line** | **Expression level** [%**TSP**]**^*^** |
| --- | --- | --- |
| 1 | 38 | 0.0078 |
| 2 | 72 | 0.0146 |
| 3 | 36 | 0.0284 |
| 4 | 86 | 0.0333 |
| 5 | 2 | 0.1098 |
| 6 | 80 | 0.1167 |
| 7 | 84 | 0.1187 |
| 8 | 83 | 0.1210 |
| 9 | 8 | 0.1276 |
| 10 | 85 | 0.1326 |
| 11 | 34 | 0.1327 |
| 12 | 37 | 0.1340 |
| 13 | 101 | 0.1380 |
| 14 | 9 | 0.2278 |
| 15 | 19 | 0.2288 |
| 16 | 25 | 0.2323 |
| 17 | 15 | 0.2413 |
| 18 | 40 | 0.2419 |
| 19 | 53 | 0.2469 |
| 20 | 31 | 0.2472 |
| 21 | 89 | 0.2609 |
| 22 | 14 | 0.2614 |
| 23 | 95 | 0.2621 |
| 24 | 52 | 0.2659 |
| 25 | 7 | 0.2694 |
| 26 | 21 | 0.2825 |
| 27 | 13 | 0.2838 |
| 28 | 24 | 0.2905 |
| 29 | 30 | 0.2974 |
| 30 | 54 | 0.3045 |
| 31 | 70 | 0.3079 |
| 32 | 28 | 0.3183 |
| 33 | 27 | 0.3247 |
| 34 | 16 | 0.3286 |
| 35 | 22 | 0.3330 |
| 36 | 26 | 0.3345 |
| 37 | 4 | 0.3371 |
| 38 | 62 | 0.3427 |
| 39 | 51 | 0.3473 |
| 40 | 74 | 0.3476 |
| 41 | 73 | 0.3520 |
| 42 | 33 | 0.3570 |
| 43 | 55 | 0.3580 |
| 44 | 29 | 0.3605 |
| 45 | 75 | 0.3818 |
| 46 | 79 | 0.3891 |
| 47 | 59 | 0.3962 |
| 48 | 78 | 0.4036 |
| 49 | 67 | 0.4426 |
| 50 | 60 | 0.4704 |

^*^ The quantity of recombinant protein (H5-ELP) present in each sample was calculated using the standard protein (anti-hTNFα-VHH-ELP [19]).

Table S4. H5 accumulation levels in transgenic tobacco leaves

| **Number** | **Transgenic line** | **Expression level** [%**TSP**]**^*^** |
| --- | --- | --- |
| 1 | 18 | 0.0038 |
| 2 | 8 | 0.0040 |
| 3 | 17 | 0.0087 |
| 4 | 16 | 0.0097 |
| 5 | 20 | 0.0099 |
| 6 | 3 | 0.0111 |
| 7 | 9 | 0.0114 |
| 8 | 4 | 0.0121 |
| 9 | 25 | 0.0128 |
| 10 | 7 | 0.0131 |
| 11 | 2 | 0.0139 |
| 12 | 10 | 0.0148 |
| 13 | 15 | 0.0152 |
| 14 | 23 | 0.0158 |
| 15 | 1 | 0.0171 |
| 16 | 19 | 0.0201 |
| 17 | 11 | 0.0397 |
| 18 | 6 | 0.0413 |

^*^ The quantity of recombinant protein (H5) present in each sample was calculated using the standard protein (anti-hTNFα-VHH-ELP [19]).

Table S5. H5-HFBI accumulation levels in transgenic tobacco leaves

| **Number** | **Transgenic line** | **Expression level** [%**TSP**]**^*^** |
| --- | --- | --- |
| 1 | 33 | 0.0044 |
| 2 | 24 | 0.0045 |
| 3 | 2 | 0.0049 |
| 4 | 25 | 0.0057 |
| 5 | 34 | 0.0063 |
| 6 | 15 | 0.0065 |
| 7 | 29 | 0.0074 |
| 8 | 30 | 0.0077 |
| 9 | 16 | 0.0092 |
| 10 | 9 | 0.0092 |
| 11 | 51 | 0.0093 |
| 12 | 20 | 0.0100 |
| 13 | 32 | 0.0103 |
| 14 | 42 | 0.0104 |
| 15 | 10 | 0.0107 |
| 16 | 17 | 0.0107 |
| 17 | 23 | 0.0108 |
| 18 | 14 | 0.0109 |
| 19 | 8 | 0.0110 |
| 20 | 50 | 0.0115 |
| 21 | 6 | 0.0124 |
| 22 | 3 | 0.0126 |
| 23 | 13 | 0.0127 |
| 24 | 46 | 0.0132 |
| 25 | 43 | 0.0133 |
| 26 | 35 | 0.0135 |
| 27 | 22 | 0.0164 |
| 28 | 39 | 0.0167 |
| 29 | 41 | 0.0167 |
| 30 | 37 | 0.0176 |
| 31 | 38 | 0.0178 |
| 32 | 21 | 0.0183 |
| 33 | 36 | 0.0186 |
| 34 | 40 | 0.0198 |
| 35 | 47 | 0.0217 |
| 36 | 11 | 0.0303 |
| 37 | 18 | 0.0466 |
| 38 | 19 | 0.0488 |

^*^ The quantity of recombinant protein (H5-HFBI) present in each sample was calculated using the standard protein (anti-hTNFα-VHH-ELP [19]).

Table S6. H5-ELP accumulation levels in transgenic tobacco leaves

| **Number** | **Transgenic line** | **Expression level** [%**TSP**]**^*^** |
| --- | --- | --- |
| 1 | 72 | 0.0468 |
| 2 | 84 | 0.0585 |
| 3 | 90 | 0.0603 |
| 4 | 75 | 0.0722 |
| 5 | 38 | 0.1029 |
| 6 | 97 | 0.1029 |
| 7 | 81 | 0.1055 |
| 8 | 2 | 0.1185 |
| 9 | 74 | 0.1214 |
| 10 | 16 | 0.1227 |
| 11 | 83 | 0.1307 |
| 12 | 68 | 0.1340 |
| 13 | 64 | 0.1445 |
| 14 | 61 | 0.1540 |
| 15 | 36 | 0.1767 |
| 16 | 65 | 0.1795 |
| 17 | 76 | 0.1870 |
| 18 | 77 | 0.1895 |
| 19 | 78 | 0.1895 |
| 20 | 30 | 0.1942 |
| 21 | 79 | 0.1974 |
| 22 | 73 | 0.1996 |
| 23 | 102 | 0.2007 |
| 24 | 14 | 0.2065 |
| 25 | 6 | 0.2134 |
| 26 | 37 | 0.2137 |
| 27 | 92 | 0.2146 |
| 28 | 82 | 0.2221 |
| 29 | 101 | 0.2271 |
| 30 | 87 | 0.2310 |
| 31 | 89 | 0.2314 |
| 32 | 71 | 0.2477 |
| 33 | 5 | 0.2643 |
| 34 | 7 | 0.2848 |
| 35 | 67 | 0.3418 |
| 36 | 99 | 0.3494 |
| 37 | 85 | 0.3537 |
| 38 | 17 | 0.3660 |
| 39 | 96 | 0.3846 |
| 40 | 94 | 0.3884 |
| 41 | 62 | 0.4080 |
| 42 | 39 | 0.4090 |
| 43 | 70 | 0.4591 |

^*^ The quantity of recombinant protein (H5-ELP) present in each sample was calculated using the standard protein (anti-hTNFα-VHH-ELP [19]).
